# Nonnegative principal component analysis for mass spectral serum profiles and biomarker discovery

**DOI:** 10.1186/1471-2105-11-S1-S1

**Published:** 2010-01-18

**Authors:** Henry Han

**Affiliations:** 1 Department of Mathematics and Bioinformatics, Eastern Michigan University, Ypsilanti MI 48109, USA

## Abstract

**Background:**

As a novel cancer diagnostic paradigm, mass spectroscopic serum proteomic pattern diagnostics was reported superior to the conventional serologic cancer biomarkers. However, its clinical use is not fully validated yet. An important factor to prevent this young technology to become a mainstream cancer diagnostic paradigm is that robustly identifying cancer molecular patterns from high-dimensional protein expression data is still a challenge in machine learning and oncology research. As a well-established dimension reduction technique, PCA is widely integrated in pattern recognition analysis to discover cancer molecular patterns. However, its global feature selection mechanism prevents it from capturing local features. This may lead to difficulty in achieving high-performance proteomic pattern discovery, because only features interpreting global data behavior are used to train a learning machine.

**Methods:**

In this study, we develop a nonnegative principal component analysis algorithm and present a nonnegative principal component analysis based support vector machine algorithm with sparse coding to conduct a high-performance proteomic pattern classification. Moreover, we also propose a nonnegative principal component analysis based filter-wrapper biomarker capturing algorithm for mass spectral serum profiles.

**Results:**

We demonstrate the superiority of the proposed algorithm by comparison with six peer algorithms on four benchmark datasets. Moreover, we illustrate that nonnegative principal component analysis can be effectively used to capture meaningful biomarkers.

**Conclusion:**

Our analysis suggests that nonnegative principal component analysis effectively conduct local feature selection for mass spectral profiles and contribute to improving sensitivities and specificities in the following classification, and meaningful biomarker discovery.

## Background

With the rapid advances in proteomics, mass spectroscopic serum proteomic pattern diagnostics has been appearing as a revolutionary cancer diagnostic paradigm. However, this technology still remains as an important field in clinical research study rather than a clinical routine testing [1]. There are many issues to be resolved to realize the routine clinical testing. Asides from the issues like data reproducibility and quality control [2], one essential issue prevents it going beyond clinical research study sets is that there is no robust supervised learning algorithm to classify proteomic patterns with high sensitivities and specificities. Although there is an urgent need to predict cancer molecular patterns with high accuracies to support clinical decisions, it is still a challenge for oncologists and computational biologists to achieve a high-performance classification due to the special characteristics of mass spectral data.

The mass spectral data has large or even huge dimensionalities. It can be represented by a *n *× *m *matrix, each row of which represents the ion intensity values of all biological samples in investigation at a mass charge ratio (m/z); each column of which represents the ion intensity values of a single biological sample at different m/z values. Each raw data can be called a *pseudo-gene *since it is similar to a gene in a gene expression dataset. Generally, the total number of m/z ratios is in the order of 10^4^~10^6 ^and the total number of biological samples is on the magnitude of hundreds, i.e., the number of variables is much greater than the number of biological samples. Although there are a large number of m/z ratios in a mass spectral profile, only a small number of them have meaningful contributions to the data variations.

Many feature selection algorithms are employed to reduce the protein expression data dimensions, remove noise, and extract meaningful features before further classification or clustering. These algorithms include two-sample t-tests, principal component analysis (PCA), independent component analysis (ICA), nonnegative matrix factorization (NMF) and their different variants [3-5]. PCA may be the most employed among them for its simplicity. It projects data in an orthogonal subspace generated by the eigenvectors of the data covariance matrix. The maximum variance direction-based subspace spanning guarantees the least information loss in the feature selection. However, as a holistic feature selection algorithm, PCA can only capture global features instead of local features [6]. The global and local features contribute to the global and local characteristics of data that are responsible for interpreting the global and local behavior of data respectively. The standard PCA by nature can not extract local features. This not only leads to difficulty in interpreting each principal component (PC) intuitively, but also causes some difficulty in achieving high-performance proteomic pattern discovery, because only the features interpreting global behavior of data are used to train a learning machine (e.g., a support vector machine (SVM) [7]). Since redundant global features may be involved in training, it will decrease the generalization of the learning machine and increase the risk of misclassifications or over-fitting. Moreover, the global data characteristics of a cancer or normal pattern are generally similar because they follow the same protein profiling mechanism. This can be easily verified by the direct visualization of mass spectral profiles. In other words, the local data characteristics play a key role in distinguishing cancer and normal proteomic patterns.

One reason for the holistic mechanism of PCA is that its data representation is not 'purely-additive'. The linear combination to calculate each PC contains both positive and negative weights. The positive and negative weights are likely to partially cancel each other in the linear combination. In fact, weights representing contributions from local features are more likely to be cancelled out because of their frequencies. This partial cancellation may directly lead to missing captures of local features for each loading vector. Another reason for the global nature of PCA is that it lacks some level sparse representation. Each loading vector receives contributions from all input variables in the linear combination. Changes in one variable will inevitably affect all loading vectors globally.

Imposing nonnegativity constraints on PCA can remove the partial cancellations in the linear combinations and make data representation consist of only additive components, i.e., restrict all entries of the input data and each PC as nonnegative items. Adding nonnegativity on PCA is also motivated by proteomic pattern discovery itself. The mass spectral profiling data is generally represented as a positive matrix naturally. It is reasonable to require its corresponding dimension-reduction data to be positive or at least nonnegative to maintain data locality in the feature selection for the sake of pattern discovery. Furthermore, imposing nonnegativity constraints on PCA also leads to the sparse representation of loading vectors.

In this study, we present a nonnegative principal component analysis (NPCA) algorithm and propose a nonnegative principal component analysis based support vector machine algorithm (NPCA-SVM) for high-performance proteomic pattern discovery. We demonstrate its algorithm superiority by comparing it with six peer classification algorithms on four benchmark mass spectral serum datasets. In addition, we present an effective biomarker discovery approach based on nonnegative principal component analysis.

This work is evolved from our previous naïve work on protein expression classification [8]. However, our current work has the following major advances/differences compared to the previous work. 1. A robust gradient learning scheme is developed for nonnegative principal component analysis and a complete nonnegative principal component analysis based support vector machine algorithm is proposed rigorously. 2. The optimal orthogonal parameter selection method is discussed and an empirical parameter choice approach is given. In addition, we also give a method to set the sparseness control parameter. 3. In addition to including previous three datasets and regenerating all simulation results, we include a new dataset: colorectal data in the experiment. Moreover, a new comparison algorithm: ICA-SVM is included in the simulation. 4. A nonnegative component principal analysis based filter-wrapper biomarker discovery by employing Bayesian t-test based filtering is proposed and its biomarker discovery results for the ovarian and colorectal data are analyzed and visualized. 5. The major global feature selection methods are presented and the two key concepts: global and local features are defined and their impacts in classifications are discussed. 6. We dropped all figures, and tables, and redundant results (e.g. over-fitting analysis about comparison algorithms) from the previous work.

## Methods

Nonnegative principal component analysis is an extension of the classic PCA algorithm by imposing it with nonnegativity constraints to capture data locality in the feature selection. Let *X *= (*x*_1_, *x*_2_,⋯ *x*_*n*_), *x*_*i *_∈ ℝ^*d *^be a zero mean dataset, the nonnegative PCA can be formulated as a constrained optimization problem to find maximum variance directions under nonnegative constraints as follows,(1)

where *U *= [*u*_1_, *u*_2_,⋯*u*_*k*_], *k *≤ *d *is a set of nonnegative PCs. The square Frobenius norm for a matrix A is defined as . The penalty parameter *α *≥ 0 controls the orthonormal degree of each loading vector. The principal component matrix *U *is a near-orthonormal nonnegative matrix, i.e., *U*^*T*^*U*~*I *Calculating the gradient of the objective function with respect to *U*, we have the learning scheme: *U*(*t*+1) = *U*(*t*) - *η*(*t*)∇_*U*_*J*(*t*)/||∇_*U*_*J*(*t*)||, *U *≥ 0 where ∇_*U*_*J *(*U*, *α*) = (*U*^*T*^*X*)*X*^*T *^+ 4*α*(*I*-*U*^*T*^*U*)*U*^*T *^and *η*(*t*) is the t time level iteration step size. We select *η*(*t*) = 1 in the implementation to avoid an expensive trust region search. This is equivalent to finding the local maximum of function  under the constraints *u*_*sl *_≥ 0 on the scalar level (*s *= 1, 2⋯*d*, *l *= 1, 2⋯*n*), where coefficients *c*_2_, *c*_1 _and *c*_0 _are parameters to be determined in the local optimum finding. The final principal component matrix *U *is a collection of nonnegative roots of function *f *(*u*_*sl*_). Calculating the stationary points and collecting the coefficients of *u*_*sl *_and , we obtain the following coefficients *c*_2_, *c*_1 _(The coefficient *c*_0 _= -*kα *has no contribution to the entries of the PC matrix).(2)(3)

The nonnegative principal component analysis complexity is *O*(*dkn *× *N*), where *N *is the total iterations needed to meet the algorithm termination threshold ||∇_*U*_*J*(*t*)|| ≤ 10^-4 ^in the implementation. Other authors also proposed a similar approach to solve a nonlinear optimization problem induced by a nonnegative sparse PCA [9], where two penalty parameters were employed to control the orthonormality and sparseness of the PC matrix. However, an additional sparseness control parameter will increase the risk of algorithmic convergence difficulty with the increasing of the parameter values [10].

We propose a nonnegative principal component analysis based classification algorithm to achieve the high-performance proteomic pattern prediction. The algorithm employs nonnegative principal component analysis to obtain the nonnegative representation of each sample in a low-dimensional, purely-additive subspace spanned by meta-variables. A meta-variable is a linear combination of the intensity values of the pseudo-genes in a mass spectral profile. The nonnegative representation for each sample is denoted as a meta-sample, which is the locality-preserved prototype of the original biological sample with low dimensionalities. Then, a classification algorithm, which is chosen as a support vector machine algorithm (SVM) [7] in this study, is applied to the meta-samples to gain classification information. Given a protein expression training dataset consisting of *d *biological samples across *n *pseudo-genes and their label information: , where *X *= [*x*_1_, *x*_2_⋯*x*_*d*_]^*T *^*x*_*i *_∈ ℝ^*n *^and *c *= [*c*_1_, *c*_2_⋯*c*_*d*_]^*T*^, *c*_*i *_∈ {-1, 1}, the NPCA-SVM algorithm finds the meta-samples *U *= [*u*_1_, *u*_2_⋯*u*_*d*_]^*T*^, *U *∈ ℝ^*d*×*k*^, *k *≤ *d *≪ *n*, by the described steepest descent method. Then, an optimal separating hyperplane *O*_*h*_: *w*^*T*^*u *+ *b *= 0 in ℝ^*d *^is computed to attain the maximum margin between the '-1' and '1' types of the meta-samples. This is equivalent to solving the following quadratic programming problem in ℝ^*d*^,(4)

Given an unknown type sample *x' *∈ ℝ^*n*^, the NPCA-SVM learning machine employs the following decision rule to determine its class type: , where *u*_*i*_, *u' *∈ ℝ^*d *^are the meta-samples of samples *x*_*i*_, *x' *computed from nonnegative principal component analysis respectively. The vector *α *= [*α*_1_, *α*_2_⋯ *α*_*d*_] ≥ 0 is the solution of the dual problem of the QP in Eq. (4) and *k*(*u*_*i*_•*u*') is a kernel function for the support vector machine, which maps these meta-samples into a same-dimensional or high-dimensional feature space. We only focus on the linear and '*rbf*' kernels for their popularity [7].

We employ a sparse-coding approach to improve the sparseness for each meta-sample. The sparseness of a nonnegative vector *v *= [*v*_1_, *v*_2_⋯*v*_*n*_]^*T*^, *v*_*i *_≥ 0, *i *= 1, 2⋯*n*, is defined as a ratio between 0 and 1:  according to the relationship of two norms [6]. A large sparseness *δ*_*v *_indicates less number of positive entries in the vector *v *Extreme cases *δ*_*v *_= 1 or *δ*_*v *_= 0 indicate that there is only one entry or all entries are equal in *v *respectively. The sparse coding of a meta-sample , *i *= 1, 2⋯*d*, *k *≤ *d *≪ *n *seeks to find a nonnegative vector *v *∈ ℝ^1×*k *^such that , and *δ*_*v *_achieving a specified sparseness value. In other words, for each loading vector  in the nonnegative PC matrix, the nearest nonnegative vector *v *on behalf of *L*_1 _and *L*_2 _distances is found to achieve a specified sparseness *δ*_*v*_. It is equivalent to calculating the nonnegative intersection point between a hyperplane  and a hypersphere  such that the sparseness degree . Since the traditional approach to find the optimal *α *is computationally expensive [10], we select *α *∝ *d *in practice because of ||*U*^*T*^*U*|| = *d*^2 ^in the extreme case where *U *is the identity matrix, if there is no further sparse coding applied to loading vectors. Otherwise, we select . Also because data sparseness is a by-product of the nonnegativity constraints in Eq. (1), we usually select the sparseness degree for each nonnegative principal component as *δ*_*v *_≤ 0.5.

We implement the NPCA-SVM algorithm under the 100 trials of 50% holdout cross validations (HOCV), i.e., 100 sets of training and testing data are generated randomly for each dataset. The final classification rate, sensitivity and specificity are the average values of these measures among the 100 trials of classifications. To improve computing efficiency, the PC matrix *U *in the nonnegative principal component analysis is cached from the previous trial and used as the initial point to compute the next principal component matrix in the computation.

## Results

Four serum proteomic datasets: ovarian, ovarian-qaqc (quality assurance/quality control), liver and colorectal are included in this study [11-13], which are generated from three different profiling technologies. Table 1 provides the detailed information about the datasets.

**Table 1:** Four mass spectral serum profiles

Dataset	Technology	#m/z	#Samples
Ovarian	SELDI-TOF low resolution	15142	91 controls + 162 cancers
Ovarian-qaqc	SELDI-TOF high resolution	15000	95 controls + 121 cancers
Liver	SELDI-QqTOF high resolution	6107	176 controls + 181 cancers
Colorectal	MADLI-TOF high resolution	16331	48 controls + 64 cancers

We conducted the following preprocessing for each dataset: baseline correction, smoothing, normalization, peak identification and peak calibration by using Matlab bioinformatics toolbox 3.3. In addition, we applied the standard two-sample t-test to select 3780, 2500, 3000 and 1000 most significant pseudo-genes for the ovarian, ovarian-qaqc, liver, and colorectal data respectively before further classifications. The goal of this basic feature selection is to select approximately 10 × *d *most significant features for each input dataset *X *∈ ℝ^*d*×*n *^before classification. We compared the nonnegative principal component analysis based support vector machine algorithm with the six peers: k-NN, SVM, PCA-SVM, NMF-SVM, ICA-SVM and PCA-LDA algorithms in terms of average classification rates, sensitivities, and specificities under 100 trials of 50% HOCV. Detailed information about the algorithms: LDA, NMF and ICA algorithms can be found in [14,6,5]. In the NPCA-SVM algorithm, we set the orthonormal control *α *= 10, the sparseness for each loading vector *δ*_*v *_= 0.20, and *k *= *d *- 1 in the NPCA feature selection due to *n *≫ *d*

We showed the average performance of the seven algorithms in terms of average classification rates, sensitivities, specificities, and their corresponding standard deviations in Table 2. We did not include performance of the SVM, PCA-SVM, ICA-SVM and NMF-SVM algorithms under the '*rbf' *kernel, because the first three encountered over-fitting and the last had lower performance under the '*rbf' *kernel than the linear kernel. We had the following observations from these results. 1) The NPCA-SVM algorithm achieved obviously leading advantages over the others. Its average specificities for the two ovarian cancer datasets reached 99%+ that was the population screening requirement ratio in the clinical diagnostics. It also achieved 98.35% average specificity for the colorectal data and 98.35% average sensitivity for the liver data. It was the only algorithm among the seven algorithms that achieved consistently leading performances for all datasets. 2) There was no over-fitting associated with the NPCA-SVM algorithm under the '*rbf*' kernel. Alternatively, it achieved exceptional sensitivities and specificities under this kernel. 3) The conventional feature selection algorithms PCA, NMF and ICA generally did not contribute to the improvements of SVM classifications.

**Table 2:** Comparisons of the seven algorithms

	Average Classifying rate (%)	Average Sensitivity (%)	Average Specificity (%)
**Ovarian**			
*npca-svm-linear*	98.94 ± 00.65	98.35 ± 01.03	99.98 ± 00.24
*npca-svm-rbf*	99.79 ± 00.35	100.0 ± 00.00	99.42 ± 00.99
*svm-linear*	99.50 ± 00.83	100.0 ± 00.00	98.63 ± 02.21
*pca-svm-linear*	99.96 ± 00.26	99.98 ± 00.17	99.93 ± 00.51
*nmf-svm-linear*	97.41 ± 00.94	99.91 ± 00.31	92.92 ± 02.50
*knn*	96.53 ± 01.57	99.28 ± 01.34	91.67 ± 03.67
*pca-lda*	99.67 ± 00.87	99.93 ± 00.38	99.21 ± 02.00
*ica-svm-linear*	99.99 ± 00.08	99.99 ± 00.12	100.0 ± 00.00
**Ovarian-qaqc**			
*npca-svm-linear*	98.70 ± 00.89	98.01 ± 01.94	99.27 ± 00.90
*npca-svm-rbf*	98.91 ± 00.98	98.11 ± 02.25	99.57 ± 00.82
*svm-linear*	96.57 ± 01.99	96.16 ± 03.52	96.97 ± 02.19
*pca-svm-linear*	97.12 ± 01.17	97.14 ± 02.16	97.94 ± 01.57
*nmf-svm-linear*	88.69 ± 03.47	92.02 ± 05.01	86.24 ± 05.67
*knn*	90.87 ± 02.92	89.99 ± 04.68	91.82 ± 04.43
*pca-lda*	97.69 ± 00.65	98.81 ± 01.68	96.99 ± 00.03
*ica-svm-linear*	97.56 ± 01.45	97.80 ± 02.46	97.41 ± 01.77
**Liver**			
*npca-svm-linear*	96.02 ± 01.35	97.68 ± 01.71	94.40 ± 02.22
*npca-svm-rbf*	97.25 ± 01.30	98.35 ± 01.67	96.20 ± 02.01
*svm-linear*	91.78 ± 02.27	92.57 ± 03.84	91.04 ± 03.76
*pca-svm-linear*	90.21 ± 01.99	90.96 ± 03.69	89.57 ± 03.56
*nmf-svm-linear*	77.76 ± 02.48	84.58 ± 05.14	71.30 ± 05.12
*knn*	76.48 ± 02.20	72.27 ± 04.60	80.80 ± 04.57
*pca-lda*	90.08 ± 02.13	91.39 ± 03.53	88.87 ± 03.95
*ica-svm-linear*	86.61 ± 02.87	87.78 ± 04.55	86.50 ± 04.86
**Colorectal**			
*npca-svm-linear*	98.14 ± 01.27	97.93 ± 02.32	98.35 ± 02.00
*npca-svm-rbf*	97.15 ± 01.07	95.81 ± 02.78	98.18 ± 02.22
*svm-linear*	96.55 ± 01.87	94.35 ± 03.47	98.26 ± 02.16
*pca-svm-linear*	93.21 ± 03.38	92.59 ± 04.68	93.89 ± 05.56
*nmf-svm-linear*	94.73 ± 03.09	92.71 ± 06.14	96.49 ± 03.45
*knn*	95.05 ± 03.17	96.17 ± 02.91	94.28 ± 05.33
*pca-lda*	94.05 ± 02.78	94.16 ± 03.74	94.01 ± 04.12
*ica-svm-linear*	96.04 ± 02.02	94.38 ± 03.66	97.39 ± 02.97

**Figure 1:**
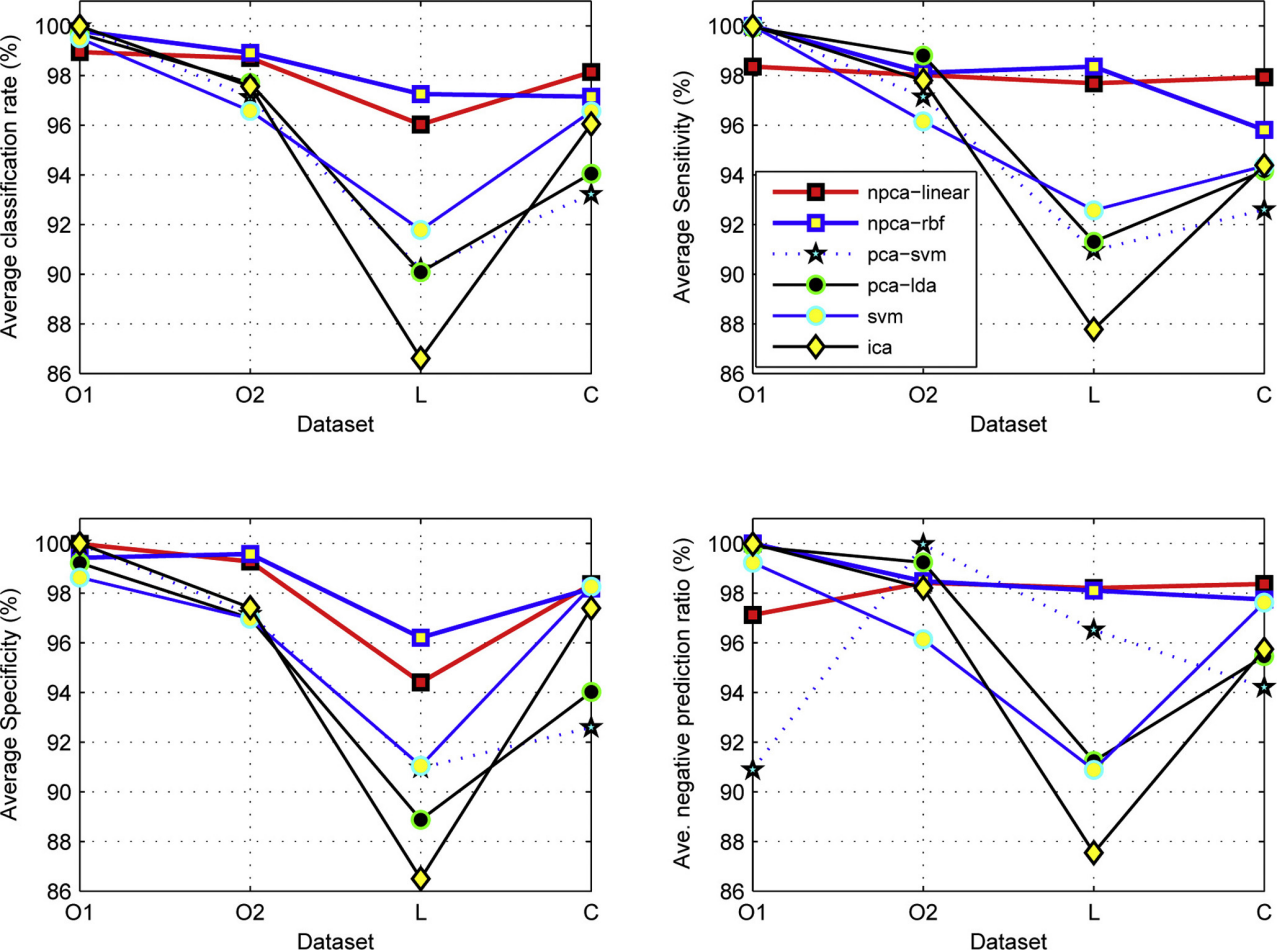
**Comparison on the five algorithm performance**. Comparison on the five algorithm performance on four datasets: 'O1' (*ovarian*), 'O2' (*ovarian-qaqc*), 'L' (*liver*), and 'C' (*colorectal*). The NPCA-SVM algorithm demonstrated leading performance over the other four algorithms.

Figure 1 compares the average classification rates, sensitivities, specificities and negative target prediction ratios of the NPCA-SVM algorithms with those of the other four algorithms: ICA-SVM, PCA-SVM, SVM and PCA-LDA. It was obvious that the NPCA-SVM algorithm with sparse coding under the '*rbf*' and '*linear*' kernels demonstrated superior or comparable performance compared with the other four algorithms.

### Biomarker discovery by nonnegative principal component analysis

In this section, we presented a nonnegative principal component analysis based filter-wrapper biomarker capturing algorithm. The Bayesian two-sample t-test [15] and nonnegative principal component analysis functioned as filters and a SVM classifier worked as a wrapper in this algorithm. Unlike other peak-selection based biomarker capturing methods [12,13], our algorithm could identify which pseudo-genes were more effective in predicting cancer patterns. The NPCA-based biomarker discovery algorithm can be described as follows. For an input mass spectral data *X *∈ ℝ^*n*×*m *^with *m *pseudo-genes and *n *biological samples, we first filter a potential biomarker set *S*_*b *_by conducting the two-sample Bayesian t-test, which is a novel approach to evaluate each pseudo-gene according to their differentially expressed levels. The potential biomarker set *S*_*b *_consists of significantly differentially-expressed pseudo-genes. For each dataset, we select at least the top 1% pseudo-genes with the smallest Bayesian factors, i.e., |*S*_*b*_| = ⌈*m *× 0.01⌉ to construct *S*_*b*_. Then, nonnegative principal component analysis (NPCA) is employed to decompose the input data: *X*^*T*^~*PU*^*T *^For each pseudo-gene, a coefficient *τ *is used to rank its contribution to all PCs. For example, the coefficient for the *i*^*th *^pseudo-gene is calculated as the weighted sum of the *i*^*th *^row in the nonnegative *P *matrix: , where  is the ratio of variance explained in the *j*^*th *^PC among the total data variance. A large coefficient value of a pseudo-gene indicates it has significant contributions to the PCs.

Each pseudo-gene in *S*_*b *_is used to train a SVM classifier under the leave-one-out cross validation (LOOCV). The first biomarker *g*_1 _is selected as the pseudo-gene with the highest accuracy. If there is more than one candidate, the pseudo-gene with the largest coefficient in NPCA-ranking will be selected. The potential biomarker set is updated by removing the selected biomarker, i.e, *S*_*b *_= *S*_*b *_- {*g*_1_}. The second biomarker *g*_2 _is selected from the current *S*_*b *_such that the SVM classifier reaches its maximum classification rate for the combination of *g*_1 _and *g*_2_. If there is more than one candidate, the pseudo-gene with the largest coefficient in the NPCA-ranking will be chosen as *g*_2_. Similarly, *S*_*b *_is updated as *S*_*b *_= *S*_*b *_- {*g*_2_}. Such a proceeding continues until the SVM classifier achieves the maximum classification accuracy with the fewest biomarkers.

**Figure 2:**
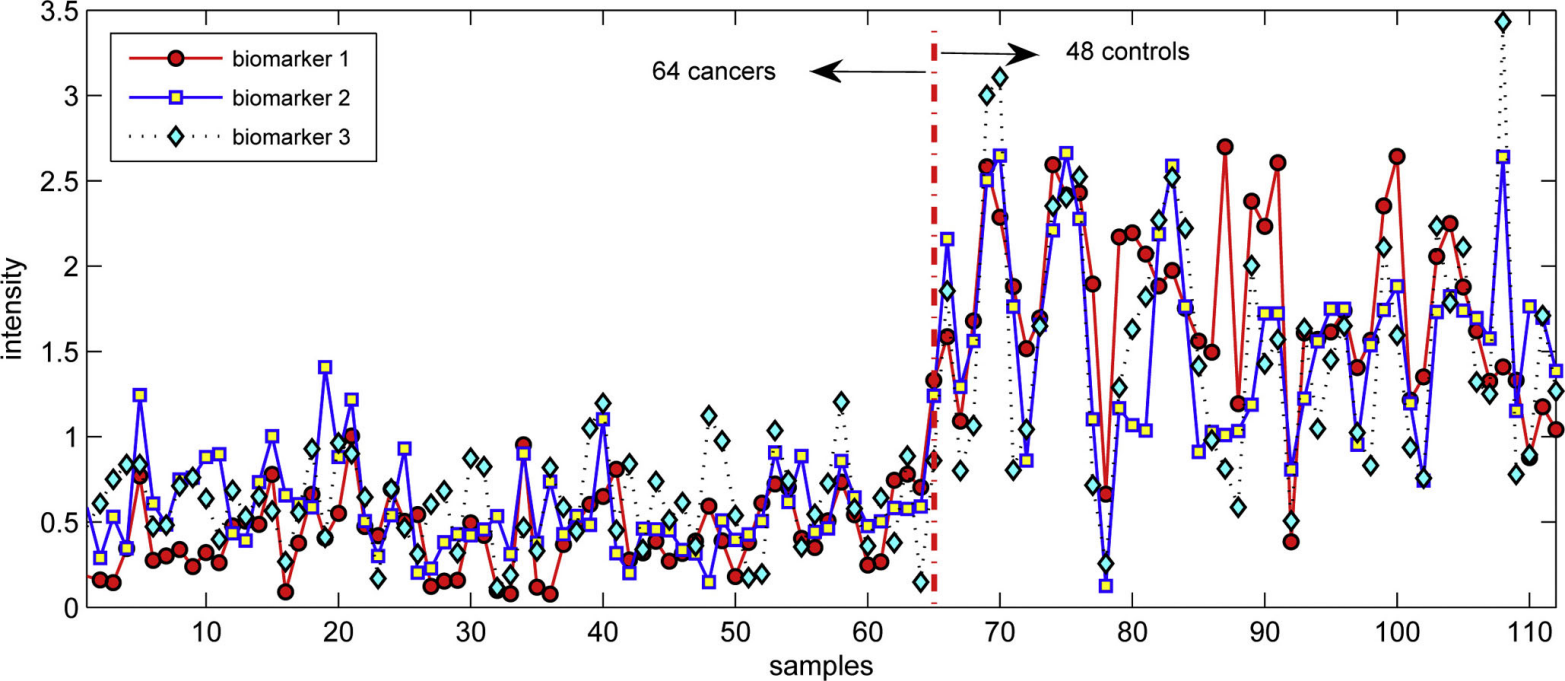
**Visualization of the colorectal samples by using three biomarkers**. The 48 control and 64 cancer samples are visualized by using the three biomarkers. Two types of samples demonstrated significantly different means and variations.

We applied the nonnegative principal component analysis based biomarker capturing algorithm to the colorectal dataset. The potential biomarker set *S*_*b *_was initialized by 200 pseudo-genes with the smallest Bayes factors. The alpha value in NPCA was set as *α *= 10 to maintain consistency with the previous classification setting. Table 3 shows the information about three biomarkers discovered for the colorectal data. The total SVM accuracy under the three biomarkers was 98.21% and the corresponding sensitivity and specificity were 95.83% and 100% respectively, which was better than the biomarker discovery results obtained in [13]. It was interesting that these biomarkers were not peaks with very large intensity values. The similar results can also be obtained by running the biomarker capture algorithms under the '*rbf*' kernel. The final SVM accuracy also reached 98.21% with three biomarkers at 970.0379, 973.1689 and 997.5336 Da. Interestingly, the biomarkers from different kernels not only shared a same pseudo-gene at 997.5336 Da, but also demonstrated a spatial coherence, i.e., they were neighbors close or very close to each other among 16331 m/z ratios in the data. It indicated that m/z ratios in the downstream interval 960-1030 Da may be more sensitive in discovering cancer patterns than others. Figure 2 visualizes all samples of the colorectal data by using the three biomarkers found under the linear kernel. It is clear that the 112 samples are partitioned into two groups: 64 cancers and 48 controls, and the two types of samples showed significantly different mean and variance values.

**Table 3:** Biomarkers captured for the colorectal data

m/z	Bayes factor	npca-coefficient	SVM ratio (%)
969.1849	7.7881e-031	-1.1205	0.9643
997.5336	1.4236e-026	-1.1571	0.9018
1016.389	7.6644e-013	1.2773	0.8152

**Figure 3:**
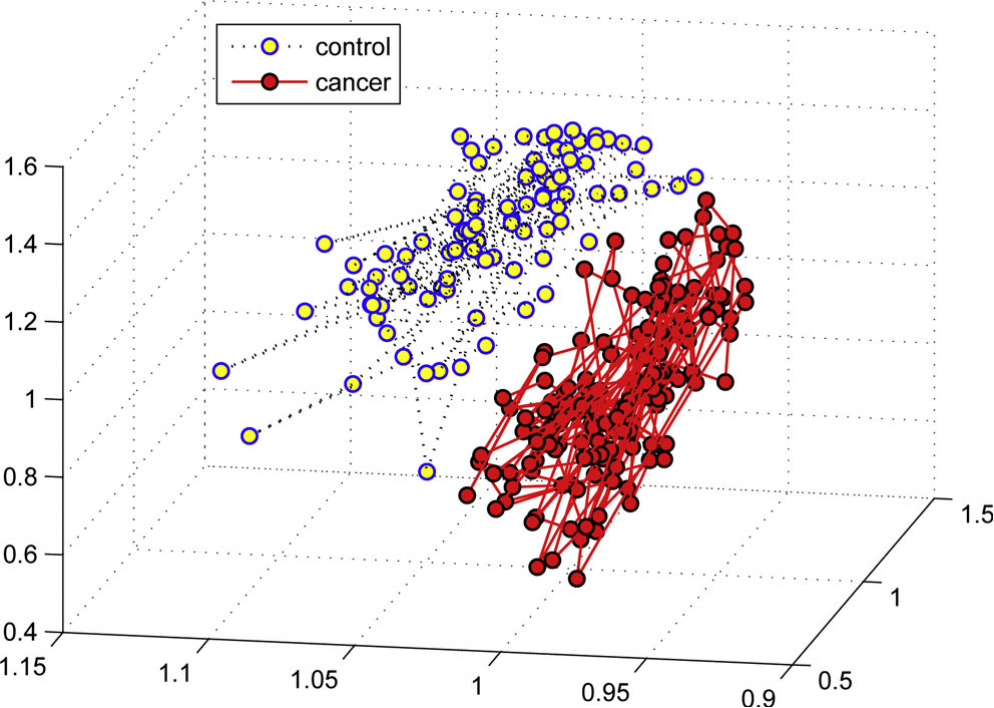
**Visualization of the ovarian samples by using three biomarkers**. The 253 ovarian samples are visualized by using the three biomarkers. The 91 control and 162 cancer samples are separated into two disjoint clusters.

Similarly, we applied this algorithm to the ovarian data and obtained 100% predication accuracy (sensitivity: 100%, specificity: 100%) from four biomarkers at m/z ratios: (0.452124, 0.000096, 0.530561, 1.276201) under the linear kernel. Moreover, The SVM classifier also achieved 99.60% accuracy, 100% sensitivity, and 98.90% specificity under the '*rbf*' kernel from three biomarkers at m/z ratios: (0.464762, 0.000096, 0.517053). Also similar to the previous case, the biomarkers discovered under different kernels illustrated spatial proximity and shared same pseudo-genes. Figure 3 visualizes all 253 samples by using the three biomarkers obtained from the '*rbf*' kernel. It was also obviously that cancer and control samples were separated clearly by the three biomarkers.

## Discussion

Although nonnegative principal component analysis has overcome the global nature of the standard PCA algorithm, and contributed to the high-performance proteomic pattern prediction and effective biomarker capture, it is an expensive algorithm with a high complexity *O*(*d*^2^*n *× *N*) compared to the classic PCA algorithm *O*(*d*^3^) for an input data *X *∈ ℝ^*d*×*n*^, *d *≪ *n*. It may require some basic feature selection preprocessing such as the two-sample t-test to avoid a large computing burden for a high-dimensional dataset. On the other hand, since the final PC matrix in nonnegative principal component analysis is computed through a fixed instead of an optimal step size in the iteration, it may miss some local optimal solutions and lead to potential convergence problems. In the following work, we plan to improve nonnegative principal component analysis (NPCA) in the following aspects. (1) We plan to employ the wavelet based multi-resolution approach to overcome the high algorithm complexity in NPCA. A wavelet transform is first employed to decompose an input data into a multi-resolution form. The nonnegative principal component analysis (NPCA) is then employed to extract the local data features from the fine level wavelet transform coefficients, which are relatively low dimensional data compared with the input protein expression data. (2) We will employ a projected-gradient algorithm [10] with a dynamic step size to improve the nonnegative principal component analysis algorithm convergence. As a local feature selection algorithm, nonnegative principal component analysis can be integrated with other state-of-the-art classification and clustering algorithms to develop a family of statistical learning algorithms. For instance, we are interested in combining it with the linear programming SVM algorithm [7] to further explore its potentials in proteomic data pattern prediction. Moreover, we will continue to investigate the applications of the NPCA-SVM algorithms in SNP, CGH array data analysis, and other related topics in future work, in addition to integrating the sparse-coding in our previous NPCA-SVM algorithm developed for gene expression profiles [16].

## Conclusion

In this work, we developed a novel feature selection algorithm, nonnegative principal component analysis, and proposed the nonnegative principal component analysis based support vector machine algorithm with sparse coding for high performance proteomic pattern discovery. We demonstrated the superiority of this algorithm by comparing it with other six peer algorithms on four proteomic datasets. In addition, we have designed a NPCA-based filter-wrapper biomarker capturing algorithm and applied it to effectively capture meaningful biomarkers for the colorectal and ovarian data. Our analysis suggests that nonnegative principal component analysis has advantages over the conventional feature selection algorithm such as PCA, ICA, and NMF in local feature selections. Although its algorithmic complexity is higher than that of widely used PCA algorithm, its nature of local feature selection contributes to the high-performance serum proteomic pattern classification and meaningful biomarker discovery.

## Competing interests

The author declares that they have no competing interests.

## Authors' contributions

HEY did all the work for this paper.
